# Using Telemedicine Strategy to Implementing Nutrition Management for Neonates After Congenital Heart Disease Surgery: A New Nutrition Management Strategy

**DOI:** 10.3389/fped.2022.918742

**Published:** 2022-06-16

**Authors:** Qi-Liang Zhang, Wen-Hao Lin, Shi-Hao Lin, Hua Cao, Qiang Chen

**Affiliations:** ^1^Department of Cardiac Surgery, Fujian Branch of Shanghai Children’s Medical Center, Fuzhou, China; ^2^Fujian Children’s Hospital, Fuzhou, China; ^3^College of Clinical Medicine for Obstetrics & Gynecology and Pediatrics, Fujian Medical University, Fuzhou, China; ^4^Fujian Key Laboratory of Women and Children’s Critical Diseases Research, Fujian Maternity and Child Health Hospital, Fuzhou, China

**Keywords:** remote nutrition management, congenital heart disease, neonates, growth and development, nutrition

## Abstract

**Objective:**

The purpose of this study was to investigate the effect of remote nutrition management on promoting the growth and development of neonates after congenital heart disease (CHD) surgery.

**Materials and Methods:**

This study retrospectively analyzed the clinical data of 32 neonates after CHD surgery who received remote nutrition management from January 2021 to July 2021 in our hospital. The clinical data of 30 neonates after CHD surgery, who did not receive remote nutrition management from June 2020 to December 2020, was used as control. The growth and development of the two groups were compared.

**Results:**

Three months after discharge, the weight, height, and weight-for-age z score (WAZ) of the intervention group was significantly higher than those of the control group. The amount of milk in the intervention group was also significantly more than that of the control group, and more neonates in the intervention group added high-energy milk or breast milk fortifier than the intervention group. The parental care ability of the intervention group was significantly higher than that of the control group. The incidence of respiratory tract infection and readmission in the intervention group was significantly lower than that in the control group.

**Conclusion:**

As a new nutrition management strategy for neonates after CHD surgery, remote nutrition management can effectively improve the nutritional status of neonates and promote their growth and development.

## Introduction

Congenital heart disease (CHD) is one of the most common congenital structural malformations, and surgery is the main treatment (1–3). With improvements in surgical techniques and intensive care, an increasing number of neonates survive after CHD surgery (4). Poor weight gain after CHD surgery is associated with an increased risk of post-operative infection and readmission and is also a risk factor for death in late infancy (5). Due to the immature organs of neonates and the injury of cardiac surgery, neonates, after CHD surgery, are more vulnerable, and severe feeding and nutrition problems are common after discharge (6, 7). Studies show that feeding difficulties, malnutrition, and growth disorders are the biggest stressors for families after discharge (8, 9).

At present, there are few studies on the feeding programs of these patients, and they mainly focus on the preoperative and perioperative periods. Few studies have focused on home nutrition management strategies for neonates with CHD surgery after discharge (10). Therefore, formulating a strategy for neonatal nutrition management with CHD surgery after discharge is essential. In recent years, telemedicine has been widely used as an educational health promotion strategy for disease management, such as diabetes, hypertension, depression, and so on (11–13). We used telemedicine to implement a new nutrition management strategy for neonates with CHD after discharge. It remotely monitors and manages the nutritional status of neonates and adjusts the nutrition program in real-time to promote their growth and development and realize nutritional catch-up.

## Materials and Methods

This study was approved by the ethics committee of our hospital and strictly adhered to the tenets of the Declaration of Helsinki. In addition, all parents of patients signed an informed consent form before the study.

This was a retrospective study. We retrospectively analyzed the clinical data of 32 neonates after CHD surgery who received remote nutrition management from January 2021 to July 2021 in our hospital. These patients were included in the intervention group. The clinical data of 30 neonates after CHD surgery who did not receive remote nutrition management from June 2020 to December 2020 were used as controls. We evaluated the effect of remote nutrition management on promoting the growth and development of neonates after CHD surgery.

Inclusion criteria: neonates undergoing CHD surgery in the neonatal period. Exclusion criteria: (1) neonates with liver failure and kidney failure; (2) combined with other severe structural malformations; (3) died after surgery or after discharge; and 4 parents of patients refused to participate in this study.

### Nutritional Management Method

We calculated energy requirements according to Leonberg et al.’s guidelines with a target caloric intake of 120–150 kcal/kg/day to ensure adequate energy intake without refeeding syndrome risk (14). Parents were asked to record the amount of milk and body weight daily. Parents were also asked to calculate patients’ daily caloric intake. The amount of milk was added according to the weight. If the patients cannot absorb enough milk and the target calorie cannot be achieved, breast milk can be fortified with a breast milk fortifier or a high-density formula milk can be used. If the patients have feeding difficulties and cannot be fed by mouth, nasal feeding should be used.

### Remote Nutrition Management After Discharge

We used WeChat (Tencent Ltd., Shenzhen, China) as the remote nutrition management tool. WeChat is the most popular mobile social media application in China, with 1.12 billion users (15). WeChat is a convenient and intuitive information exchange platform with functions such as graphics, text, audio, and video to maximize information coverage.

**FIGURE 1:**
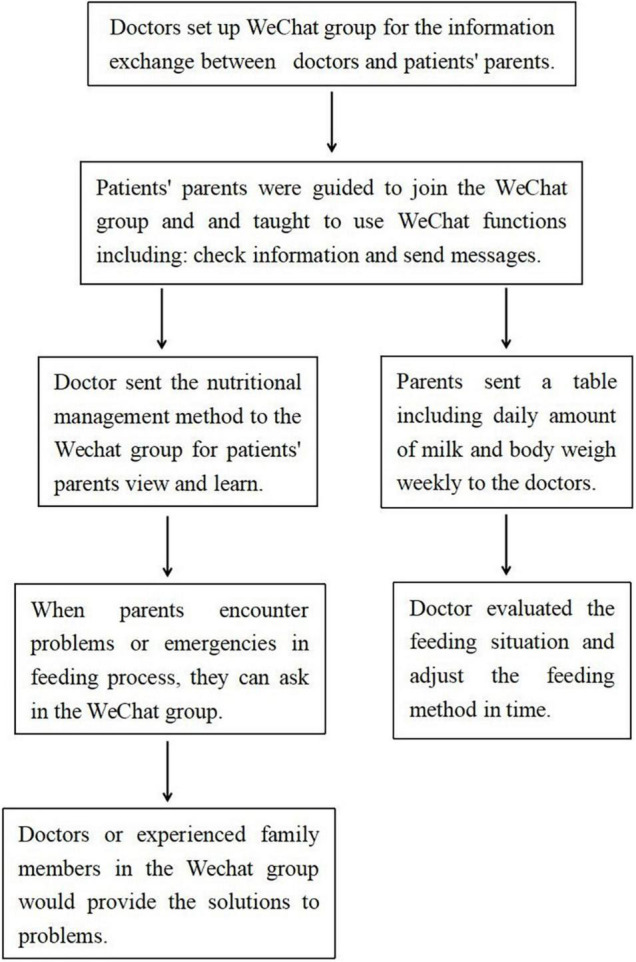
The flow chartof remote management nutrition *via* Wechat.

We set up chat groups through WeChat. At the time of discharge, parents in the intervention group were guided to join the WeChat group and taught to use WeChat functions correctly and skilfully by the doctor. Parents needed to know how to check WeChat information and send messages. It was simple and can be easily mastered. We sent the nutritional management method to the WeChat group for all parents of patients to view and learn. A doctor on our team was available to answer parents’ questions on WeChat from 18:00 to 21:00 every day. If the parents encountered problems or emergencies in the feeding process, they could also seek help from doctors or experienced family members in the WeChat group, and problems could be solved in time. Parents could also communicate with each other in the WeChat group to share their care and feeding experiences. Parents were asked to send a weekly table to the doctor *via* WeChat. The table included the amount of milk and body weight daily, so that the doctor could evaluate the feeding situation and adjust the feeding method accordingly (Figure 1).

### Traditional Nutrition Management After Discharge

The nutritional management method was printed in the manual and sent to the parents of the control group at discharge. If the situation was abnormal, they were instructed to return to the hospital for timely review. If there was an emergency, they were instructed to go to a nearby hospital in time.

All patients in our department were reviewed in the outpatient department 3 months after discharge. The data collected at the 3-month follow-up included growth and development data, as well as complication data, such as pneumonia, feeding difficulties, feeding intolerance, liver insufficiency, times of readmission, and the Family Caregiver Task Inventory (FCTI) scale.

### Family Caregiver Task Inventory Scale

The FCTI scale was used to evaluate the care ability of the parents. The scale consists of 25 items, including 5 dimensions: adapting to care roles, responding, and providing assistance, addressing personal emotional needs, assessing family and community resources, and adjusting personal life and care needs. Each entry adopts the Likert 3-grade scoring method: 0 points mean not difficult, 1 point means difficult, and 2 points mean extremely difficult. The total score on the scale is 50 points. The higher the score, the more difficulty the caregiver faces, and the lower the care ability (16).

### Statistical Analysis

The SPSS 25.0 software was used for statistical analysis. Continuous data are presented as the mean ± standard deviation (SD). The continuous data conformed to a normal distribution by the normal distribution test. Continuous data between the two groups were compared by *t*-test, and a *p*-value of <0.05 was defined as a statistically significant difference. Qualitative data between the two groups were compared by Fisher’s test, and a *p*-value of <0.05 was defined as a statistically significant difference.

## Results

The general information of patients and their parents at discharge in the two groups, including age, height, weight, weight-for-age z score (WAZ), disease, type of feeding, parents’ age, parent’s education level, family income, and family living environment, showed no statistical significance (Table 1).

**TABLE 1:** Demographic data of patients and their parents in two groups at discharge.

	Intervention group	Control group	*P*
Number	32	30	
Age (Day)	19.4 ± 6.3	20.2 ± 6.6	0.346
Weight (kg)	3.4 ± 0.4	3.3 ± 0.5	0.456
Height (cm)	47.1 ± 1.5	46.9 ± 1.7	0.405
WAZ	−2.1 ± 0.4	−2.0 ± 0.5	0.539
Disease			
Ventricular septal defect	7	6	0.995
Patent ductus arteriosus	8	9	
Pulmonary stenosis	2	2	
Aorta arch constriction	3	2	
Ventricular septal defect with patent ductus arteriosus	6	7	
Total anomalous pulmonary venous connection	2	2	
Complete transposition of great arteries	2	1	
Aortic arch interrupt	2	1	
Feeding patterns			
Nasogastric	7	6	0.925
Bottle-feeding	21	21	
Direct breastfeeding	4	3	
Age of parents (year)	39.6 ± 3.8	30.5 ± 5.2	0.306
Family income			
Low income	9	10	0.897
Middle-income	18	16	
High income	5	4	
Parents’ education level			
Under high school	6	5	0.905
High school	8	7	
Junior college	12	10	
Bachelor degree or higher	6	8	
Living condition			
Rural area	20	19	0.946
City	12	11	

*The age of premature infants was calculated after corrected gestational age.*

Three months after discharge, the comparison of feeding situation, growth, and development between the two groups showed that the weight, height, and WAZ of the intervention group were significantly higher than those of the control group. The amount of milk and patients eating high-energy milk or breast milk fortifiers in the intervention group were also significantly higher than those in the control group. The care ability of the parents in the intervention group was significantly higher than that in the control group (Table 2).

**TABLE 2:** Comparison of feeding, growth and development between the two groups 3 months after discharge.

	Intervention group	Control group	*P*
Weight (kg)	5.8 ± 0.5	5.3 ± 0.8	0.016
Height (cm)	57.7 ± 3.7	55.4 ± 2.9	0.010
WAZ	−0.7 ± 0.5	−1.3 ± 0.8	0.006
Adding high-energy milk or breast milk fortifier	15	6	0.025
Amount of milk	142.2 ± 14.7	115.0 ± 13.2	0.000
FCTI score of parents	20.9 ± 5.9	24.5 ± 4.6	0.011

During the follow-up period 3 months after discharge, the incidence rates of respiratory tract infection and readmission in the intervention group were significantly lower than those in the control group. There was no significant difference in the incidence of feeding intolerance, cardiac insufficiency, or arrhythmia between the two groups. No necrotizing enterocolitis, liver insufficiency, renal insufficiency, or gastrointestinal bleeding occurred in the two groups (Table 3).

**TABLE 3:** Comparison of complications between the two groups during 3 months follow-up time.

	Intervention group	Control group	*P*
Respiratory tract infection	3	9	0.040
Feeding intolerance	7	4	0.379
Necrotizing enterocolitis	0	0	–
Readmission	1	6	0.036
Liver insufficiency	2	1	0.593
Renal insufficiency	0	0	–
Gastrointestinal hemorrhage	0	0	–
Cardiac insufficiency	0	1	–
Arrhythmology	3	2	0.696

## Discussion

Malnutrition and disturbance of growth have always been common and serious problems in infants with CHD, and the more complex and severe CHD is, the more serious these problems are (17). For patients who need surgical treatment in the neonatal period, their condition is critical before surgery, and after surgical trauma, extracorporeal circulation trauma, and post-operative fluid restriction treatment, patients face serious malnutrition at discharge (18, 19). Homecare for neonates after CHD surgery is a daunting task (20). Nutrition and feeding problems are the biggest source of stress for parents (8, 9). Malnutrition and growth disorders are highly prevalent and associated with poor clinical outcomes (21). However, at present, there is no optimal nutritional management strategy for infants with CHD after discharge, especially for neonates with severe CHD.

In recent years, with the rapid development of medical information technology, telemedicine has been widely used in the home management of chronic diseases, which is conducive to improving home care ability, improving the prognosis of patients, and reducing the incidence of complications (22, 23). To solve the feeding and nutrition problem of neonates with CHD surgery, promote weight, growth and development catch-up, and reduce the incidence of complications, we performed nutrition management *via* remote medicine, which can extend high-quality medical services from hospitals to families, children with remote management of feeding, and implementation of nutritional support. We remotely managed the patients’ feeding and implemented nutritional support. WeChat is the most widely used medical media in China, so WeChat was chosen as the media for remote nutrition management. Through remote nutrition management, we can regularly remind families to learn about feeding programs and monitor their feeding and weight gain. When parents have problems learning about feeding programs or feeding, we also guided them remotely to learn and improve. In these ways, parental care ability in the intervention group was significantly better than that in the control group.

Weight gain, or even weight catch-up, mainly depends on the amount of milk consumed and the energy density of milk, therefore, improving the amount of milk and energy density of milk promotes maximum weight gain (24). Neonatal gastrointestinal function after CHD surgery is often inadequate, and they are prone to feeding-related complications, such as vomiting, diarrhea, and abdominal distension (25, 26). Many parents of these neonates lack care ability, skill, and experience. To prevent feeding-related complications, they cannot add milk, do not dare to add high-energy milk or breast milk fortifier, and feed in small amounts and many times. This often makes neonates perpetually hungry, leading to poor sleep, which is not conducive to weight gain, growth, or development. Remote nutrition management can regularly monitor the feeding situation, correct bad feeding habits, and help neonates reach the target milk volume and target calories as often as possible. For those who did not meet the standard, we guided the addition of high-energy milk or breast milk fortifier to improve energy density. Although adding high-energy milk or breast milk fortifier potentially increases complications of feeding intolerance, (27) we instructed parents to closely observe the related complications. In cases of abdominal distention, diarrhea, constipation, etc., probiotics, glycerine enema, or Montmorillonite powder was used in the early stage for symptomatic treatment. There was no increase in the incidence rate of feeding intolerance complications in the intervention group compared with the control group in this study. Our study showed that after remote nutrition management, the feeding volume in patients who received high-energy milk or breast milk fortifier in the intervention group was significantly better than those in the control group. As a result, the weight and WAZ of the intervention group were significantly higher than those of the control group.

Gastrointestinal and feeding complications, such as dysphagia, feeding intolerance, and necrotizing enterocolitis, are common problems of neonates after CHD surgery and persist for a long time after discharge (25, 26). Gastrointestinal and feeding problems have a significant impact on weight gain, growth, and development, and they are also associated with post-operative complications and readmission. Thirteen patients in this study had dysphagia at discharge and required nasogastric feeding. If such patients cannot be meticulously cared for during the feeding process, they are prone to vomiting and choking milk, which can easily lead to respiratory tract infections. Malnourished infants are also prone to respiratory tract infections and other complications, leading to readmission (28). Remote nutrition management can effectively improve the nutritional status and growth and development of infants, improve the parenting ability of children, and reduce the incidence of respiratory complications and readmission rates.

This study has some limitations. First, this study was a single-center retrospective study with small sample size. Second, due to unstable internet support, poor patients could not participate in the study.

## Conclusion

Remote nutritional management as a new nutritional management strategy for neonates with CHD surgery after discharge can effectively improve nutritional status and promote growth and development.

## Data Availability Statement

The original contributions presented in this study are included in the article/supplementary material, further inquiries can be directed to the corresponding author.

## Ethics Statement

This study was approved by the ethics committee of our hospital and strictly adhered to the tenets of the Declaration of Helsinki. There were no human subjects involved in this work. In addition, all patients’ guardians signed an informed consent form before the study.

## Author Contributions

Q-LZ designed the study, acquired and interpreted the data, and drafted the manuscript. S-HL analyzed and interpreted the data. W-HL acquired and analyzed the data. HC had made substantial contributions to conception and design. QC was involved in the analyzes and interpretation of data, revising the manuscript, and given final approval of the version to be published. All authors contributed to the article and approved the submitted version.

## Conflict of Interest

The authors declare that the research was conducted in the absence of any commercial or financial relationships that could be construed as a potential conflict of interest.

## Publisher’s Note

All claims expressed in this article are solely those of the authors and do not necessarily represent those of their affiliated organizations, or those of the publisher, the editors and the reviewers. Any product that may be evaluated in this article, or claim that may be made by its manufacturer, is not guaranteed or endorsed by the publisher.

## Abbreviations

CHD: congenital heart disease
FCTI: family caregiver task inventory
WAZ: weight-for-age z score.
